# Bordetella hinzii bacteremia in a patient with SARS-CoV-2 infection

**DOI:** 10.1080/22221751.2022.2147276

**Published:** 2022-12-20

**Authors:** Kamilia Chahi, Christine Collienne, Ahalieyah Anantharajah, Hector Rodriguez-Villalobos, Philippe Hantson

**Affiliations:** a Department of Intensive Care, Cliniques Universitaires St-Luc, Université Catholique de Louvain, Brussels, Belgium; b Laboratory of Microbiology, Cliniques Universitaires St-Luc, Université Catholique de Louvain, Brussels, Belgium; c Louvain Centre for Toxicology and Applied Pharmacology, Université Catholique de Louvain, Brussels, Belgium

During the recent coronavirus disease (COVID-19) pandemic, a significant number of patients experienced severe bacterial and fungal co-infections with a serious impact on COVID-19 mortality [1]. This condition could also predispose to some rare opportunistic infections. We report a case of bacteremia due to a particular Bordetella species in a patient with a chronic lung disease and a recent contamination by SARS-CoV-2.

A 75-year-old man presented to the Emergency Department for asthenia, loss of appetite and progressive dyspnoea. He had a medical history of chronic obstructive pulmonary disease (Gold D). The patient was still an active smoker (4–5 cigarettes/day). He was vaccinated for Influenza and Streptococcus pneumonia but had refused vaccination against SARS-CoV-2 virus. He reported a fever at home, but there was no objective recording of body temperature. On admission, he complained mainly of abdominal pain and vomiting. Vital signs were as follows: body temperature 35° C, respiratory rate 30/min, heart rate 78/min, blood pressure 150/78 mmHg, oxygen pulse saturation (SpO2) 91% with 2 L/min. A major cachexia (43 kg body weight, body mass index 15.9 kg/m^²^) and frank signs of dehydration were noted. Lung auscultation revealed diffuse hypoventilation. The abdomen was distended, with weak bowel sounds. Blood test results (reference ranges) showed C-reactive protein 336.5 mg/L (<5.0), leukocytes 11,510 cells/mm^³^ (400–1000.10^³^), 88.2% neutrophils, lymphocytes 0.32.10^³^ (0.80–5.00.10^³^), platelets 259,000/mm^³^ (150–450.10^³^), serum creatinine 3.41 mg/dL (0.60–1.30). Urine antigenic testing was negative for *Streptococcus pneumoniae* and *Legionella pneumophila*. A SARS-CoV-2 nasopharyngeal swab sample test was positive by RT–PCR (Cepheid Xpert® Xpress SARS-CoV-2 Plus), with a high viral load (43,868,273 copies/ml). The variant was identified as Omicron BA2. The chest-X-ray examination was not impressive, with a discrete retrocardiac infiltrated and marked signs of panlobular lung emphysema. No sputum could be obtained for bacteriological analysis, and urine culture was sterile. We started the patient on dexamethasone 6 mg/day for 10 days; remdesevir was not considered due to impaired renal function. Empirical treatment with amoxicillin/clavulanic acid was initiated for bacterial pneumonia. By the second hospital day, the patient was transferred to the Intensive Care Unit (ICU) for a worsening of his neurological status associated with a progression of respiratory acidosis. Arterial blood gas analysis revealed: pH 7.27, pCO2 74 mmHg, pO_2_ 99 mmHg while the patient received high-flow nasal cannula oxygen therapy (60 L/min). Chest-X-ray examination appeared unchanged. Bacterial growth was detected in the both aerobic bottles (BACTEC Plus Aerobic/F, Becton Dickinson Diagnostic Systems, Sparks, MD, United States) drawn on hospital admission after 2 days and 9 hours and 3 days and 2 hours of incubation, respectively. The anaerobic blood culture bottles from patients remained negative. Gram revealed small rod-shaped Gram-negative microorganisms. The positive bottles were subcultured on Columbia 5% sheep blood agar in a 5% CO_2_ atmosphere at 35°C according to routine laboratory workflow. *Bordetella hinzii* was identified within 24 hours from the subcultures using matrix-assisted laser desorption ionization time-of-flight mass spectrometry (Microflex LT mass spectrometer and IVD MALDI Biotyper System, Bruker, Bremen, Germany) with a score of 2.24. The partial sequencing of the 16S ribosomal ribonucleic acid (rRNA) gene and Basic Local Alignment Search Tool (BLAST) analysis of the consensus confirmed the identification (>99.9% sequence identity with *Bordetella hinzii* 16S rRNA gene). Minimum inhibitory concentration (MIC) was determined by the E-test gradient method (E-test®, bioMérieux, Lyon, France) on Mueller-Hinton Fastidious agar. The antibiogram revealed that the micro-organism was resistant to amoxicillin/clavulanic acid and therapy was shifted for trimethoptrim/sulfamethoxazole (Table 1).

**Table 1.** Antimicrobial susceptibility of *Bordetella hinzii* isolate using E-test gradient method.

Antimicrobial agent	MIC (µg/mL)	Interpretation^a^
Amoxicillin/clavulanic acid	16	Resistant
Piperacillin/tazobactam	1	Sensitive
Ceftriaxone	>32	Resistant
Ceftazidime	2	Sensitive
Meropenem	0.125	Sensitive
Ciprofloxacin	2	Resistant
Trimethoprim/sulfamethoxazole	0.25	^b^

^a^ Antimicrobial susceptibility to the different antimicrobial agents was interpreted according to EUCAST Clinical Breakpoints version 12.0, PK-PD (non-species related) breakpoint:

^b^ No breakpoints were considered for trimethoprim/sulfamethoxazole MIC.

Piperacillin/tazobactam was added 3 days later after the identification of *Enterobacter cloacae* in a new blood culture. The patient did not experience cardiocirculatory dysfunction and renal function progressively improved after fluid replacement. Respiratory function also rapidly improved and the patient could leave the ICU on day 5 with 2 L/min supplemental oxygen. Transthoracic echocardiography did not suspect an abnormal aspect of cardiac valves. As a late complication, he presented a mechanical intestinal obstruction requiring laparotomy and resection of adhesions.

The *Bordetella* genus comprises 11 species, of which *Bordetella pertussis* and *B.parapertussis* are isolated from humans as the pathogen of whooping cough. *Bordetella bronchiseptica* is rarely pathogenic in humans, but isolated cases of pneumonia or bacteremia have been recently reported as coinfection of severe SARS-CoV-2 [2,3].

*B. hinzii* is a strictly aerobic gram-negative bacillus that was first identified as a cause of respiratory illnesses, mostly rhinotracheitis, in poultry [4]. Human infection remains extremely uncommon, but could include skin infection, urinary tract infection, pneumonia, pancreatic or liver abscess, peritionitis, cholangitis and infective endocarditis, with or without bacteremia. To date, less than 20 cases of B. hinzii infection were regarded as an opportunistic complications occurring in patients with underlying conditions. It has been mentioned after HIV, malignancy, liver disease ulcerative colitis, diabetes and liver transplantation [5–9]. As in most of the cases described previously, our patient was never exposed to poultry nor birds. Recently, *B. hinzii* bacteremia was reported in a 77-year-old man who presented a more severe form of SARS-CoV-2 infection [10]. In this case, B. hinzii was also identified in the bronchoalveolar lavage. The patient was treated by vancomycin and cefepime, but the isolate had a high MIC of 64 mg/L for cefepime and was only susceptible to meropenem, levofloxacin, amikacin and gentamycin. The patient died likely from the consequence of SARS-CoV-2 infection rather than co-infection by *B. hinzii*. In another 63-year-old man with COVID-19 diagnosis, nosocomial pneumonia developed 5 days after the start of mechanical ventilation [11]. *B. hinzii* was identified from endotracheal aspirate. It showed resistance to amoxicillin, cefotaxime, aminoglycosides, and ciprofloxacine; intermediate resistance to amoxicillin/clavulanic acid and ceftazidime; and susceptibility to piperacillin/tazobactam, meropenem, and imipenem. Aspergillus fumigatus was further found in endotracheal aspirate. The patient ultimately recovered following combination therapy with piperacillin/tazobactam and voriconazole. Finally, B. hinzii pneumonia was diagnosed in a 41-year-old man three days after the start of mechanical ventilation for a severe SARS-CoV-2 infection [12]. The strain was susceptible to piperacillin/tazobactam, imipenem, meropenem and trimethoprim/sulfamethoxazole. The patient made a favourable outcome after two weeks of meropenem therapy.

In our patient, the exact source of bacteremia was not identified, with as possible hypothesis respiratory tract colonization in an immunocompromised patient by SARS-CoV-2 infection or intestinal translocation following ileus. In the literature, most of the patients recovered from *B. hinzii* infection after appropriate antimicrobial therapy.

Finally, the protective role of COVID-19 vaccination against such opportunistic infections cannot be extrapolated from isolated case reports, even if it can be assumed that vaccines can activate innate immune responses.

In conclusion, SARS-CoV-2 infection may be added to the list of conditions predisposing to an opportunistic infection by *B. hinzii*.
